# Massive open online course: a new strategy for faculty development needs in healthcare simulation

**DOI:** 10.1186/s41077-024-00318-y

**Published:** 2024-11-20

**Authors:** Nadège Dubois, Céline Tonus, Sophie Klenkenberg, Anne-Françoise Donneau, Clément Buléon, Alexandre Ghuysen

**Affiliations:** 1 Public Health Department, Medical Simulation Center, University of Liège, Liège, Belgium; 2 Digital Tools Research and Education Support Unit, University of Liège, Liège, Belgium; 3 Biostatistics and Research Methods Center (B-STAT), Liège University, Liège, Belgium

**Keywords:** Faculty development, Training of trainers, Healthcare simulation, Engagement in learning, MOOC

## Abstract

Faculty development in medical simulation is a growing need, given the increased use of simulation-based learning in healthcare. Training of trainers is demanding and resource-consuming; therefore, there is a need for accessible, practical, and resource-saving solutions enabling efficient faculty development. For that purpose, we investigated whether a massive open online course (MOOC) could meet these challenges. This manuscript presents a MOOC, its pedagogical strategies, and its impacts on faculty development. The University of Liège Medical Simulation Center developed a MOOC with five learning units focused on the foundations of simulation training. Each unit ends with a set of theoretical and practical exercises. Our results showed knowledge and skills acquisition (objective exercise score data) among participants with a success rate of 67%, as well as high levels of engagement and confidence in learning (self-reported data). We demonstrate that a MOOC for faculty development in healthcare simulation is effective, has the potential to be an accessibility enabler, and offers an additional tool for trainer training. We recommend designing it with various asynchronous online modalities and multiplying social interactions with peers and trainers to favor trainees’ engagement and subsequent learning. Further perspectives should study blended learning strategies in faculty development and define the optimum ratio between face to face and online synchronous and asynchronous modalities.

**Graphical Abstract:**

Characteristics and advantages of MOOCs as an asynchronous online teaching tool for faculty development in healthcare simulation.

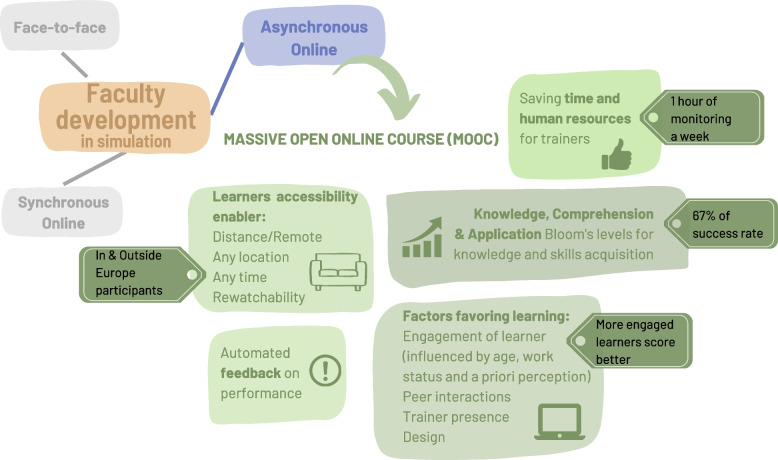

**Supplementary Information:**

The online version contains supplementary material available at 10.1186/s41077-024-00318-y.

## Background

Medical simulation in healthcare education is constantly evolving and growing. This educational tool has become central due to its relevance and potential impact on safety [1–3]. Such development was made possible through deploying and implementing many simulation centers worldwide. The need for faculty development has increased simultaneously with the need for a more qualified workforce to develop simulations, supervise them, conduct debriefings, and all the logistical organization. Faculty development can take many forms and is time, cost, and experience resource-consuming. The most popular form is face-to-face training, but there is an increasing tendency to move towards online training. Indeed, this teaching method alone covers a multitude of possibilities with synchronous, asynchronous, or blended distance learning. Although many studies of online training in faculty development exist, the results are highly variable due to the multitude of teaching modalities used, the lack of comparison with other modalities, and the many qualitative approaches [4]. Furthermore, in the medical simulation faculty development field, only a few online initiatives have been studied [5–7]. For example, initiatives like the InCITE simulation online courses or the DebriefLive virtual teaching environment have been developed [5, 6]. InCITE is designed for nursing educators. These online customized training modules meet specific needs such as interprofessional communication, leading a debriefing post-simulation and incorporating simulation into course curricula [5]. DebriefLive allows the debriefing of virtual learners after video-recorded simulation sessions [6]. Both provide effective and appropriate content, as self-reported by participants. Another pre- and post-test impact study revealed simulation knowledge gain, but there was no change in perception or intention to adopt simulation after online modules training for nursing faculty [7].

We wanted to offer a new and profitable solution to fill the gap between the growing need for educators and the realistic limited and resource-consuming increase in the number of educators while maintaining the quality of faculty development. This advancing simulation practice article aims to present our MOOC (massive open online course) as a new educational tool, its advantages, and also the impact observed in terms of knowledge and skills acquired regarding healthcare simulation faculty development as the effectiveness of asynchronous online training is unknown.

## Main text

### MOOCs characteristics

MOOCs have emerged as an easy, fast, decentralized, and large-scale way to disseminate knowledge asynchronously while allowing learners’ assessment. A recent review provides the significant roles of MOOCs in healthcare education and practice, including increasing public health literacy, providing continuing professional education, facilitating innovative teaching and learning methods, enhancing communication among international communities of patients and clinicians, obtaining large-scale data, and focusing on patient- and family-centered needs [8]. To date, four MOOCs exist to acquire expertise in healthcare simulation worldwide [9–12]. To our knowledge, no published data exists on engagement or acquisition of knowledge and skills among learners who have followed these MOOCs.

Although an online solution, the MOOC is distinguished by many aspects. In terms of scheduling, MOOCs offer active sessions, meaning that the content is available for a few successive months (called a session) over a year. Sessions can then be held every year or at other intervals, depending on demand and the availability of providers. This mode of delivery offers advantages for both learners and educators. At the learner level, MOOCs impact accessibility notably through no fees requested and organizational flexibility in both timetable (from anywhere at any time) and pace of learning (fully asynchronous online) [13]. Rewatchability through the possibility of repeatability is also an advantage [14]. These features are significant drivers for learning and learner engagement. At the educator level, MOOCs mean saving time, costs, and resources [15] on a larger scale than simple online training. Although the creation of the material takes time, running a MOOC session (i.e., having an online presence for announcements and answering questions or requests on the discussion forum) only requires a few hours of monitoring per week. Unlike online training, which requires time and resources for each session, from the second session of a MOOC, we observe a time saving since only running time remains. Scalability is also important since MOOCs can reach many learners (thousands) simultaneously or with repeated sessions [13, 14]. For all these reasons, the MOOC is an excellent solution to meet the needs of educators. We, therefore, developed a MOOC, implemented it for several years, and evaluated its effectiveness.

### MOOC setup, running and data collection

Creating the MOOC required 42 working days of three simulation and techno-pedagogy experts. Running the MOOC during a session requires 1 h of weekly monitoring by one simulation expert. It is composed of a series of units addressing the essential concepts of medical simulation: (1) introduction to medical simulation, (2) errors and human factors analysis, (3) types and structure of simulation, (4) debriefing, and (5) pedagogical strategy. Educational content is presented via various tools, such as talking head videos with illustrations, animation videos, demonstration videos, and interviews. Indeed, reviews of the literature dealing with faculty development indicate that one of the key features of effective programs is the use of a diversity of educational methods within an intervention, and that unidimensional intervention seems less effective [4, 16]. After each unit, exercises (set in different medical and nonmedical contexts) are proposed, ranging from MCQs to case or video analysis. Automatic pre-coded feedback and corrections are provided to learners, allowing them to become aware of their success and their errors. Feedback is known to be an essential element in teaching effectiveness and contributes to trainees’ achievement [17, 18]. Important MOOC culture themes are also present, such as social media mentality, instructor engagement, and peer interactions through a forum, which is a space open to discussion and questions between simulation experts and/or participants [13]. Although asynchronous, the instructor engagement can be defined as an online presence via interactions on the discussion forum as mentioned above, weekly email posts and announcements, and his repeated presence in videos such as the talking head videos throughout the MOOC modules [13].

Our MOOC “La simulation médicale: à vous de jouer!” is hosted on the FUN-MOOC platform [19] and is available for free, in French, once a year for 4 months. No prerequisites are needed to follow this MOOC. It is intended for anyone who wants to learn more about medical simulation, from healthcare students to professionals. Additional resources, such as benchmark articles in the field, are available for those wishing to gain further knowledge.

**Fig. 1:**
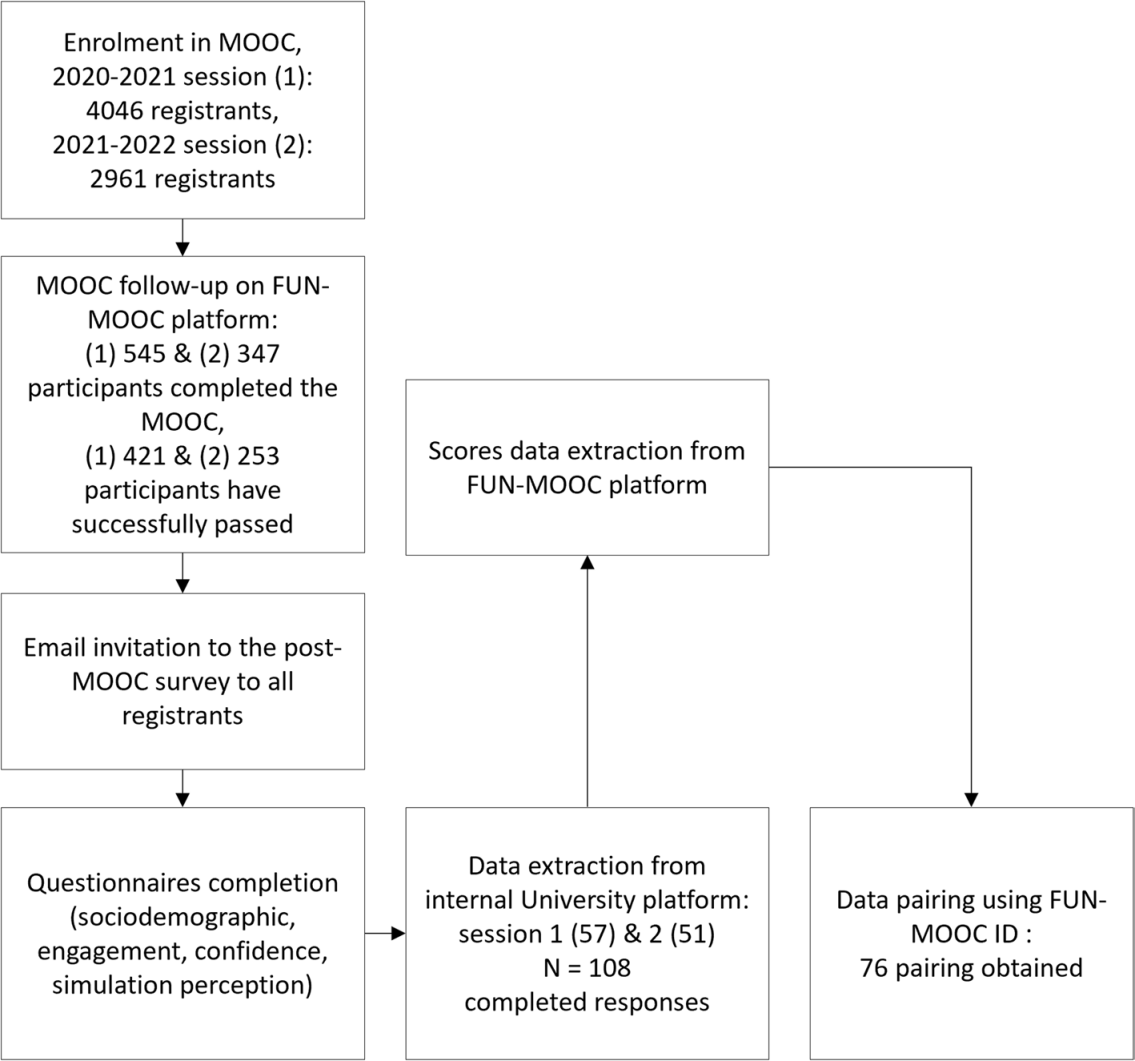
Flowchart design of the study and data collected

The first session was released from October 2020 to January 2021, and then additional sessions were held at the same period in subsequent years. We only collected data from the first two sessions of our MOOC (session 1 from October 2020 to January 2021 and session 2 from October 2021 to January 2022) and emailed the enrolled participants at the end of these two sessions. Voluntary participants answer online post-MOOC surveys. We correlated the results with the complete performance scores obtained via the FUN-MOOC platform when we successfully paired the data (Fig. 1).

The impact of the intervention was measured at two levels: first, objective data about performance (knowledge and skills) obtained in the exercises on the FUN-MOOC platform (called unit scores between 0 and 1 and weighted average score with a pass threshold of 0.7), and second, self-reported data (post-MOOC online survey) including (1) sociodemographic data, (2) engagement of participants measured using TEL (technology-enhanced learning) engagement scale [20], (3) their confidence in learning, and (4) their perception of simulation before and after completion of this MOOC. A full description of the data collection tools was used, and the statistical analysis carried out can be found in Additional file 1.

### Findings and comments

All detailed results can be found in the Additional file 2.

In our sample of survey respondents, half (51%) were over 40 years old, 79% were from Europe, and most of the respondents (83%) were professionally active. Accessibility being an important factor for us, we studied the distribution of MOOC participants in our sample and across all those registered for the first two sessions of the MOOC. We observed that for MOOC sessions 1 and 2, we have reached 60% and 64% of European respondents (excluding Belgium) and 25% and 30% of respondents from outside Europe, respectively (Table 1).

**Table 1:** Descriptive statistics of participants’ origin

Origin	Study sample (*N* = 108)	MOOC session 1 (*N* = 3852)^a^	MOOC session 2 (*N* = 2734)^a^
Belgium (%)	19 (17.6)	597 (15.5)	164 (6)
Europe (excluding Belgium) (%)	66 (61.1)	2292 (59.5)	1750 (64)
Outside Europe (%)	23 (21.3)	963 (25)	820 (30)

^a^Available data

**Fig. 2:**
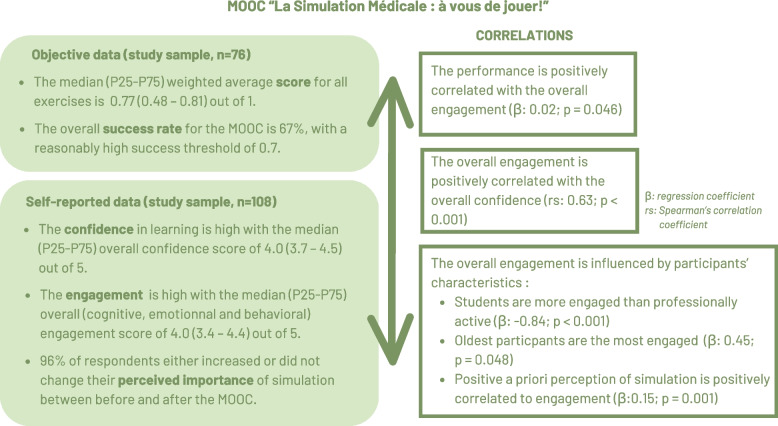
Main results observed on the study sample. All detailed results can be found in the Additional file 2

In the present work, we intended to explore the first three levels of Bloom’s taxonomy (knowledge, comprehension, and application) and reach the first two levels of Kirkpatrick’s taxonomy (reaction and learning) [21, 22] together with the evaluation of knowledge and skills acquisition in studying the impact of our MOOC. Our main result shows that the overall success rate for the MOOC was 67%, with a reasonably high success threshold of 0.7. The median scores for each unit are around 0.70 (out of 1), and the weighted average score is 0.77 (Fig. 2). These scores are associated with success in various exercises of increasing complexity, including theoretical MCQs, case analyses, video analyses, and the creation of advocacy-inquiry, allowing us to say that our MOOC is highly effective in acquiring knowledge and skills. Asynchronous online training courses such as online modules or MOOCs offer a wide range of ways of assessing the acquisition of knowledge and skills through objective data collection [7]. However, it is important to remember that scores depend not only on learning but also on the nature of the exercises. The exercises must be adapted to the pedagogical objectives, varied and multiple, and cannot in themselves represent the success of a trainer training program as a whole.

Our other findings demonstrate high levels of confidence and engagement from the participants (Fig. 2). The median confidence levels are comparable between the different units and are all equal to 4 (on a scale from 1 to 5). The engagement subtypes (emotional, cognitive, behavioral) and overall engagement indicated that the median engagement levels are high and comparable since they are all around 4 (on a scale from 1 to 5). Overall confidence was correlated with the level of engagement in our sample. Engagement in the educational environment can be defined as the quality of the efforts made by learners towards learning activities that directly contribute to desired outcomes [23]. Engagement is an important part of the learning process [24], and should be promoted, especially in online asynchronous training. Indeed, it promotes satisfaction, a sense of community, and, most importantly, retention and learning outcomes [24–26]. Emotional engagement reflects satisfaction and how a learner is willing to utilize resources. Cognitive engagement relates to the goal self-set and how he will be immersed in it, while behavioral engagement relates to physical interactions and active participation in proposed resources [27]. We observed that the engagement subtypes were positively correlated with each other. Joshi and colleagues also demonstrated this with other TELs such as MOOC, iBook, or online courses [20, 28]. However, no correlation was observed between the levels of engagement and teaching resources (MOOC, eBook, and screencast) [29]. Participant-related factors can influence engagement [30, 31]. In our sample, students were more engaged than professionally active participants for a given age category. The oldest participants were the most engaged if the professional status was fixed. In turn, it has been shown that learners’ motivation influences their engagement with TEL, and that engagement influences learning and future performance [24]. It was the case with our MOOC, where overall engagement was positively correlated with performance, indicating that more engaged individuals get better scores. In faculty development, we will probably deal with working professionals rather than more engaged students. However, the engagement will be favored by the fact that they want to train in medical simulation voluntarily or professionally.

We asked learners to share their perception of the importance of simulation in the education of health professionals and the healthcare system before and after the completion of the MOOC. Before the completion of the MOOC, the majority of respondents reported simulation to be important. This may have affected the recruitment of respondents and the results since the respondents were already interested in the subject. This is demonstrated by the significant and positive correlation we have observed between the a priori perception and each type of engagement. Ninety-six percent of respondents either increased their perception or did not change their opinion between the two surveys. Furthermore, only 4% of respondents indicated less importance after completion of the MOOC than before (Fig. 2).

### Limits

We should mention some limitations to our MOOC experience. First, at the study level, as with all surveys, we cannot exclude the fact that the people who answered the post-MOOC survey may be more involved in their learning and may not represent the entire population enrolled in the MOOC. Targeting an interested audience, like in the context of faculty development, should maximize compliance.

Second, at the educational tool level, (1) the risk of participants dropping out can be limited by targeting a specific audience. (2) Once the content is built, modifications require a major effort, and awareness of this aspect is important. The basic theoretical concepts in pedagogy and simulation are immutable and can be found in this type of content. The novelties, the various examples, and experience sharing should be found in other teaching methods. (3) There was a lack of real-time interactivity, although automated feedback and asynchronous interactions with teachers through forums were present in our MOOC. Feedback or debriefing allows for reflection on personal practices, which is essential to the learning process [17, 32].

### Perspectives

One of the solutions to the real-time interactivity question, and a perspective for this work, would be to carefully design MOOC by multiplying the teaching modalities in our MOOC, including those with direct interactions like live lectures, and to maximize interaction, even asynchronous, with trainers and peers that supported the promotion of learning. Peer-to-peer interaction enables social media mentality, learning through forum discussions, and knowledge construction and metacognition through peer evaluation [13, 33]. Unlike face-to-face training, peers’ interactions can be tremendous, as there is no limit to the participant’s number or the time they can devote to it. Although asynchronous, the trainer’s interaction is essential in creating a relationship. This is achieved through the trainer’s repeated presence in the videos and discussions on the forum. Another solution is to use MOOC as one tool among others and move towards a blended learning (BL) approach while considering the properties and advantages inherent to the various methods to maximize learners’ engagement and learning [34]. This BL solution is often proposed in the literature, particularly as a solution of choice for train-the-trainers programs in the health and social care field [35]. Systematic reviews and meta-analysis in education show that BL leads to trainees’ satisfaction and engagement, is more effective than non-BL for knowledge and skills acquisition, and improves abilities like critical thinking and clinical behavior. All agree that the level of blended still needs to be investigated [35–37]. BL remains a challenge, which requires a strong pedagogical framework basis, specific resources or devices, and user skills for teachers and trainees [36]. Table 2 summarizes the literature recommendations and our experience designing a MOOC to maximize engagement and learning.

**Table 2:** Recommendations for the design of MOOCs to promote engagement and learning

*Topics*	*Recommendations*	*References*
Focus and target audience	• Choose a subject that appeals to a large enough audience. There must be a need for	[4]
Creation resources	• Have a team of experts in techno-pedagogy and experts in medical simulation, particularly in the training of trainers	
Content	• Create stable content for theoretical or practical subjects rather than constantly evolving • Build on solid foundations, referenced in the literature, gold standards, and good practice guides • Divide the subject logically into chapters or modules	[37]
Format	• Use various educational tools (e.g., talking head video with illustrations, animation video, demonstration video, interview, tutorial, online discussion or chat, shared article, or live lecture) • Provide links to key resources in the field • Keep content online for some time to allow for rewatchability	[4] [14]
Assessment	• Assessment should be done by chapter or module • Assessments should be appropriate to the content (knowledge and skills type), of increasing complexity, and varied in form (e.g., MCQ, case analysis, video analysis, question about an article) • Include feedback in assessments (e.g., automatic pre-coded feedback)	[38] [16]
Social media mentality, interaction	• Allowing exchanges via a discussion forum or a chat gives people the opportunity to give their opinion by posting comments, voting on comments, or just reading comments and discussions	[4, 13]
Peer interaction	• Allow interaction between peers via the forum, creating a community • Use proper peer review exercises	[13, 32]
Presence of trainer	• Promote the instructor’s online presence through the presence in videos, forums, weekly emails, or announcement emails. Direct interaction is not mandatory or required by trainees	[4, 13]
Others	• Self-assess our MOOCs and improve them if necessary • Integrating MOOCs into blended learning program	[34–36]

A perspective for this work and future research should be to compare training methods like face-to-face training with different synchronous and asynchronous online ones to accurately identify MOOC’s pertinence among the others and define the ideal teaching blend for medical simulation faculty development that gives trainees a satisfactory level of knowledge and experience and saves trainers time.

## Conclusion

We reported that using a MOOC can be an effective strategy for faculty development in healthcare simulation and has the potential to be an accessibility enabler, saving time and resources for trainers and trainees. In addition, in our experience, using a MOOC for faculty development in simulation enables significant knowledge and skills acquisition associated with a high level of engagement. Their design should be carefully thought out to maximize engagement and learning. Furthermore, they enable the widespread dissemination of expertise, with easy access to experts in the field.

We believe that MOOCs are powerful tools that enrich the trainer’s toolbox.

## Supplementary Information

Additional file 1: Methods & questionnaires.

Additional file 2: Supplementary results. Supplementary Table 1. Descriptive statistics of the sociodemographic data for the study sample. Supplementary Table 2. Descriptive statistics of score (performance) variables. Supplementary Table 3. Descriptive statistics of confidence and engagement variables. Supplementary Table 4. Spearman’s correlations between engagement variables (*N* = 107). Supplementary Table 5. Associations between confidence and grades / engagement. Supplementary Table 6. Influence of sociodemographic variables and a priori perception of simulation on engagement variables (multivariate linear regressions) (*N* = 103). Supplementary Table 7. Influence of socio-demographic data, overall engagement, and a priori perception of simulation on scores (multivariate beta regression, *N* = 73). Supplementary Fig. 1. Changes in perception of the importance of simulation before and after completing the MOOC (*n* = 103). (The perception of simulation was the sum of the perception of the importance of simulation in the education of healthcare professionals and of perception of the importance of simulation in the healthcare system).

## Data Availability

The datasets used and/or analyzed during the current study are available from the corresponding author on reasonable request.

## Abbreviations

MOOC: Massive open online course
TEL: Technology-enhanced learning
MCQs: Multiple-choice questions
BL: Blended learning
