# Supplementary material for: Massive open online course: a new strategy for faculty development needs in healthcare simulation
Source: Adv Simul (Lond). 2024 Nov 20;9:44. doi: 10.1186/s41077-024-00318-y (PMC11577793; doi:10.1186/s41077-024-00318-y)
Supplement: Supplementary file 1 — Additional file 1: Methods & questionnaires. [file 41077_2024_318_MOESM1_ESM.docx]

**Additional file 1 : Methods & questionnaires**

- 1. **Study Methods description**

We followed the SQUIRE-EDU ((Standards for QUality Improvement Reporting Excellence in Education) report approach [1].

Context

Our MOOC “*La simulation médicale: à vous de jouer!*” is hosted on the FUN-MOOC platform [2], and is accessible for free, in French, once a year for four months. The first session was released from October 2020 to January 2021, and then additional sessions were held at the same period in subsequent years. No prerequisites are needed to follow this MOOC. It is intended for anyone who wants to learn more about medical simulation, from healthcare students or professionals to casuals. The FUN-MOOC platform and we spread the word through newsletters and social networks.

We collected data from the first two sessions of our MOOC and emailed the enrolled participants at the end of these two sessions. We invited them to participate in the study and answer online post-MOOC surveys, which are available two months after each session. Among the respondents to the post-MOOC survey, we only kept the complete answers per survey. We correlated the results with the complete performance scores obtained via the FUN-MOOC platform when we successfully paired the data.

Intervention

The MOOC was created by the equivalent of three people, simulation and techno-pedagogy experts, for 42 working days. Running the MOOC during a session requires one hour of weekly monitoring by one simulation expert. It is divided into five units that address (1) introduction to medical simulation, (2) errors and human factors analysis, (3) types and structure of simulation, (4) debriefing, and (5) pedagogical construction. After each unit, participants completed a series of exercises, ranging from MCQs to case or video analysis. A discussion forum was available to discuss any topic or question with a teacher, expert in medical simulation, or other participants.

Study of the intervention

The impact of the intervention was measured at two levels: objective data about performance (knowledge and skills) obtained in the exercises on the FUN-MOOC platform, and self-reported data (post-MOOC online survey) including socio-demographic data, engagement of participants, their confidence in learning and their perception of simulation before and after completion of this MOOC.

Measures

*Performance measures* consisted of scores for each of the five-course units, each ranging between 0 and 1 arising from all the unit's exercises. A weighted average of these scores was considered the overall performance measure for the MOOC. The pass threshold has been set at 0.7.

*Self-reported data*: Participants filled out online surveys, via an internal University platform. For engagement, the TEL engagement scale was used [3]. The internal validity of this questionnaire was measured with a Cronbach's coefficient of 0.867. These 19 items, with a 5-point Likert scale, measure learners’ engagement and allow us to differentiate three types of engagement: emotional, cognitive, and behavioral engagement [4]. To measure their confidence in learning, learners were asked their level of confidence (from 1: not at all confident in my learning this objective to 5: very confident in my learning this objective) for each objective related to a unit. The set of objectives related to a specific unit provides an average confidence score for that unit (ranging from 1 to 5). The importance (from 1: Not at all important to 5: Very important) that learners placed on simulation in the education of health professionals and in the healthcare system before (“a priori perception”) and after this MOOC was collected.

Data analysis

Qualitative variables were summarized with counts and percentages. The median and interquartile range (P25-P75) were displayed for quantitative variables.

Internal consistency of the different parts of the survey was evaluated using Cronbach's α coefficients and correlations between subscales.

Associations between socio-demographic data, engagement, confidence variables, and grades were first assessed using bivariate analyses. Nonparametric Kruskal-Wallis tests were applied to investigate socio-demographic variables’ influence on quantitative variables. Relationships between ordinal and/or quantitative variables were assessed using non-parametric Spearman’s correlation coefficients. Multivariate linear regressions were then used to explain engagement scores and overall confidence using socio-demographic and other relevant explanatory variables. A multivariate beta regression was applied to explain the weighted average grade.

As the performance measures were missing for 32 subjects (29.63%), homogeneity chi-squared tests of socio-demographic variables were applied to assess the representativeness of the subset of participants for whom the performance measures were available. It should also be noted that, out of the 108 respondents, 103 (95.37%) answered all survey items, whereas five respondents (4.63%) presented missing values for at least one item. These subjects were then not considered in the analyses involving the respective variables. Statistical significance is achieved at 95% confidence (p-value significance < 0.05). All tests are two-sided. The statistical software used is R version 4.1.2.

Ethical considerations

The ethical committee of the University of Liège Medicine Faculty approved this study with the reference number 2021/85. All participants gave informed consent to participate in the post-MOOC study. We collected performance data retrospectively via the platform by following the "general conditions of use and the treatment of the personal data" of FUN-MOOC. We used the ID code from FUN-MOOC to correlate all the data via pseudo-anonymization.

References

1. Ogrinc G, Armstrong GE, Dolansky MA, Singh MK, Davies L. SQUIRE-EDU (Standards for QUality Improvement Reporting Excellence in Education): Publication Guidelines for Educational Improvement. Acad Med. 2019;94:1461–70.

2. France Université Numérique. FUN-MOOC: La simulation médicale : à vous de jouer ! https://www.fun-mooc.fr/fr/cours/la-simulation-medicale-vous-de-jouer/. Accessed 24 Jan 2024.

3. Pickering JD, Swinnerton BJ. Exploring the Dimensions of Medical Student Engagement with Technology-Enhanced Learning Resources and Assessing the Impact on Assessment Outcomes. Anat Sci Educ. 2019;12:117–28.

4. Trowler V. Student engagement literature review. 2010.

- 1. **Sociodemographic questionnaire** (provided in French as in the MOOC, translated into English in this appendix)

Données **socio-professionnelles */ Sociodemographic data***

| - Age (-20 / 20-30 / 31-40 / +41) */ Age (-20 / 20-30 / 31-40 / +41)* |
| --- |
| - Pays d’origine */ Country of origin* |
| - Sexe (F / M) */ Sex (F/M)* |
| - Identifiant Fun-mooc */ Fun-mooc ID* |
| - Activité : étudiant / professionnellement actif / les 2 / retraité  *- Activity: student / professionally active / both / retired* |
| - Domaine étude/professionnel : (Médical / Paramédical / Enseignement / Autre)  *- Field of study/profession: (Medical / Paramedical / Education / Other)* |
| - Expérience en simulation médicale : Oui / Non */ Experience in medical simulation: Yes / No* |

**1.3 Technology-enhanced learning (TEL) engagement** (provided in French as in the MOOC, translated into English in this appendix)

**Questionnaire sur l’engagement**

« Technology-enhanced learning (TEL) engagement instrument », James D. Pickering, 2019, Anatomical Sciences Education

Au regard de ce MOOC « La simulation médicale à vous de jouer ! », indiquez votre degré d’accord avec ces affirmations ; *With regard to this MOOC « La simulation médicale à vous de jouer ! », please indicate your level of agreement with the following statements;*

1 : Pas du tout d'accord, 2 : Pas d'accord, 3 : Ni d'accord, ni pas d'accord, 4 : D'accord, 5 : Tout à fait d'accord

*1: Strongly disagree, 2: Disagree, 3: Neither agree nor disagree, 4: Agree, 5: Strongly agree*

| Items |  |  |  |  |  |
| --- | --- | --- | --- | --- | --- |
| J’ai profité d’une série des ressources du MOOC */ I have enjoyed a series of MOOC resources* | 1 | 2 | 3 | 4 | 5 |
| Je me suis engagé fortement dans les ressources du MOOC */ I am strongly involved in the MOOC resources* |  |  |  |  |  |
| J’ai aimé utiliser les ressources du MOOC */ I enjoyed using the MOOC resources* |  |  |  |  |  |
| Je me réjouissais d’utiliser les ressources du MOOC dans mon cours */ I looked forward to using the MOOC resources in my course* |  |  |  |  |  |
| Je recherchais activement dans les ressources du MOOC pour soutenir mon apprentissage */ I actively searched the MOOC resources to support my learning* |  |  |  |  |  |
| J’ai consacré suffisamment de temps au MOOC */ I spent enough time on the MOOC* |  |  |  |  |  |
| J’ai utilisé les ressources du MOOC pour consolider mes connaissances et réviser après les cours (les modules) */ I used the MOOC resources to consolidate my knowledge and revise after the lessons (modules)* |  |  |  |  |  |
| J’ai utilisé les ressources du MOOC pour m’aider à réaliser mes objectifs d’apprentissage */ I used the resources in the MOOC to help me achieve my learning objectives* |  |  |  |  |  |
| Je m’étais fixé des objectifs d’apprentissage avant d’apprendre avec les ressources du MOOC */ I had set myself learning objectives before learning with the MOOC resources* |  |  |  |  |  |
| J’avais prévu d’utiliser les ressources du MOOC pour mon apprentissage */ I had planned to use the MOOC resources for my learning* |  |  |  |  |  |
| J’avais planifié mon apprentissage avec l’utilisation des ressources du MOOC (planning de l’utilisation) */ I had planned my learning with the use of the MOOC resources ("planning for use")* |  |  |  |  |  |
| J’ai terminé toutes les tâches du MOOC que j’ai commencées */ I have completed all the MOOC tasks I started* |  |  |  |  |  |
| J’ai utilisé les ressources du MOOC pour préparer le cours/les exercices */ I used the MOOC resources to prepare the course/exercises* |  |  |  |  |  |
| J’ai relié ce que je savais déjà au contenu du MOOC */ I have linked what I already knew to the content of the MOOC* |  |  |  |  |  |
| J’ai (re)pensé à ce que j’avais appris après avoir suivi le MOOC */ I (re)thought about what I had learned after completing the MOOC* |  |  |  |  |  |
| J’ai pris des notes lorsque j’utilisais le MOOC */ I took notes when using the MOOC* |  |  |  |  |  |
| J’ai écouté avec attention lorsque j’utilisais les ressources du MOOC */ I listened carefully when using the MOOC resources* |  |  |  |  |  |
| J’ai passé en revue toutes les ressources du MOOC en détail */ I looked at all the resources in the MOOC in detail* |  |  |  |  |  |
| Je suis retourné voir les ressources du MOOC quand je ne comprenais pas un sujet */ I went back to the MOOC resources when I didn't understand a subject* |  |  |  |  |  |

**1.4 Level of confidence** (provided in French as in the MOOC, translated into English in this appendix)

Questions concernant leur **confiance perçue dans leurs apprentissages** (v-à-v des objectifs pédagogiques du MOOC (14)) */ Questions about their perceived confidence in their learning (in relation to the MOOC's pedagogical objectives (14))*

Au regard des différents objectifs pédagogiques du MOOC listés ci-dessous, quel est votre degré de confiance concernant votre apprentissage : */ With regard to the different learning objectives of the MOOC listed below, how confident are you in your learning?*

1 : pas du tout confiant dans mon apprentissage de cet objectif

2 : peu confiant dans mon apprentissage de cet objectif

3 : Ni confiant, ni pas confiant dans mon apprentissage de cet objectif

4 : confiant dans mon apprentissage de cet objectif

5 : Très confiant dans mon apprentissage de cet objectif

*1: not at all confident in my learning of this objective*

*2 : Not very confident in my learning of this objective*

*3 : Neither confident nor not confident in my learning of this objective*

*4 : confident in my learning for this objective*

*5 : Very confident in learning this objective*

| Unit | Items / Objectifs |  |  |  |  |  |
| --- | --- | --- | --- | --- | --- | --- |
| U1 | Savoir décrire ce qu’est la simulation médicale ? */ Describe what medical simulation is?* | 1 | 2 | 3 | 4 | 5 |
| U2 | Comprendre l’impact des facteurs humains dans l’apparition d’erreurs ? */ Understand the impact of human factors on the occurrence of errors?* |  |  |  |  |  |
| U2 | Comprendre l’importance des points clé CRM dans la gestion du travail d’équipe/ de crise ? */ Understand the importance of CRM key points in teamwork/crisis management?* |  |  |  |  |  |
| U2 | Pouvoir analyser la survenue d’un incident et ses différentes facettes */ Be able to analyse the occurrence of an incident and its various aspects* |  |  |  |  |  |
| U3 | Connaitre les différentes modalités de simulation / Be familiar with the different simulation modalities |  |  |  |  |  |
| U3 | Pouvoir expliquer l’importance de la sécurité psychologique dans l’apprentissage */ Be able to explain the importance of psychological safety in learning* |  |  |  |  |  |
| U3 | Comprendre le déroulement d’une séance complète de simulation et le rôle des différentes phases */ Understand how a complete simulation session unfolds and the role of the different phases* |  |  |  |  |  |
| U4 | Connaitre les différentes étapes du débriefing et leurs rôles */ Be familiar with the different stages of debriefing and their roles* |  |  |  |  |  |
| U4 | Comprendre l’intérêt du débriefing avec bon jugement */ Understand the value of debriefing with good judgement* |  |  |  |  |  |
| U4 | Pouvoir construire un advocacy-inquiry sur base de faits observés ou entendus */ Be able to construct an advocacy-inquiry based on facts observed or heard* |  |  |  |  |  |
| U5 | Connaitre les étapes de création d’une formation */ Know the steps involved in creating a training course* |  |  |  |  |  |
| U5 | Connaitre les étapes de création d’un scénario de simulation */ Be familiar with the steps involved in creating a simulation scenario* |  |  |  |  |  |
| U5 | Comprendre la relation entre les objectifs pédagogiques et la modalité de simulation à utiliser */ Understand the relationship between educational objectives and the simulation modality to be used* |  |  |  |  |  |
| U5 | Pouvoir construire un scénario de simulation adapté au public cible et aux objectifs pédagogiques poursuivis */ Be able to construct a simulation scenario adapted to the target audience and the educational objectives pursued* |  |  |  |  |  |

**1.5 Perceived importance of simulation-based education** (provided in French as in the MOOC, translated into English in this appendix)

Questions concernant leur **vision de la simulation médicale** */ Questions concerning their vision of medical simulation*

Quel est le degré d’importance que vous accordez aux items ci-dessous : */ How important are the following items to you?*

1 : Pas du tout important

2 : Peu important

3 : Ni peu important, ni vraiment important

4 : Important

5 : Très important

*1: Not at all important*

*2: Not very important*

*3: Neither not very important nor really important*

*4 : Important*

*5: Very important*

- - AVANT ce MOOC, comment perceviez-vous l’importance de la simulation médicale dans l’**éducation** des professionnels de la santé ? */ BEFORE this MOOC, how did you perceive the importance of medical simulation in the education of healthcare professionals?*
  - APRÈS avoir suivi ce MOOC, selon vous, quel est l’importance de la simulation médicale dans l’éducation des professionnels de la santé ? */ AFTER completing this MOOC, in your opinion, how important is medical simulation in the education of healthcare professionals?*
  - AVANT ce MOOC, comment perceviez-vous l’importance de la simulation médicale dans les **systèmes de soins de santé** ? */ BEFORE this MOOC, how did you perceive the importance of medical simulation in healthcare systems?*
  - APRÈS avoir suivi ce MOOC, selon vous, quel est l’importance de la simulation médicale dans les systèmes de soins de santé ? */ AFTER completing this MOOC, in your opinion, what is the importance of medical simulation in healthcare systems?*
