# Supplementary material for: Massive open online course: a new strategy for faculty development needs in healthcare simulation
Source: Adv Simul (Lond). 2024 Nov 20;9:44. doi: 10.1186/s41077-024-00318-y (PMC11577793; doi:10.1186/s41077-024-00318-y)
Supplement: Supplementary file 2 — Additional file 2: Supplementary results. Supplementary Table 1. Descriptive statistics of the sociodemographic data for the study sample. Supplementary Table 2. Descriptive statistics of score (performance) variables. Supplementary Table 3. Descriptive statistics of confidence and engagement variables. Supplementary Table 4. Spearman’s correlations between engagement variables (N = 107). Supplementary Table 5. Associations between confidence and grades / engagement. Supplementary Table 6. Influence of sociodemographic variables and a priori perception of simulation on engagement variables (multivariate linear regressions) (N = 103). Supplementary Table 7. Influence of socio-demographic data, overall engagement, and a priori perception of simulation on scores (multivariate beta regression, N = 73). Supplementary Fig. 1. Changes in perception of the importance of simulation before and after completing the MOOC (n = 103). (The perception of simulation was the sum of the perception of the importance of simulation in the education of healthcare professionals and of perception of the importance of simulation in the healthcare system). [file 41077_2024_318_MOESM2_ESM.docx]

**Additional file 2 : Supplementary results**

| **Supplementary Table 1 – Descriptive statistics of the sociodemographic data for the study sample** | | | |
| --- | --- | --- | --- |
| **Variable** | **Total (N=108)^a^** | **Subgroup (N=76)^b^** | **p-value** |
| Age (years) |  |  |  |
| ≤ 30 (%) | 28 (25.9) | 15 (19.7) | 0.621 |
| 31 – 40 (%) | 25 (23.2) | 19 (25) |  |
| > 40 (%) | 55 (50.9) | 42 (55.3) |  |
| Sex |  |  |  |
| Female (%) | 63 (58.3) | 44 (57.9) | 0.953 |
| Male (%) | 45 (41.7) | 32 (42.1) |  |
| Origin |  |  |  |
| Belgium (%) | 19 (17.6) | 12 (15.8) | 0.557 |
| Europe (outside Belgium) (%) | 66 (61.1) | 52 (68.4) |  |
| Outside Europe (%) | 23 (21.3) | 12 (15.8) |  |
| Professional status |  |  |  |
| Student (%) | 18 (16.7) | 9 (11.8) | 0.632 |
| Professionally active (%) | 75 (69.4) | 57 (75) |  |
| Student and professionally active (%) | 15 (13.9) | 10 (13.2) |  |
| Professional background |  |  |  |
| Medical (%) | 40 (37) | 30 (39.5) | 0.617 |
| Paramedical (%) | 35 (32.4) | 27 (35.5) |  |
| Education (%) | 14 (13) | 11 (14.5) |  |
| Other (%) | 19 (17.6) | 8 (10.5) |  |
| Simulation experience |  |  |  |
| Yes (%) | 66 (61.1) | 50 (65.8) | 0.517 |
| No (%) | 42 (38.9) | 26 (34.2) |  |

^a^ Total: Respondents to the post-MOOC survey (main study sample)

^b^ Subgroup: Among respondents, those whose performance data was extracted from the MOOC platform could be paired.

| **Supplementary Table 2: Descriptive statistics of score (performance) variables** | | |
| --- | --- | --- |
| **Variable** | **Median (P25 – P75)** | **Success**  **N (%)** |
| Score Unit 1 (introduction to medical simulation) | 0.77 (0.62 – 0.85) | 40 (53.0) |
| Score Unit 2 (errors and human factors analysis) | 0.78 (0.66 – 0.82) | 55 (72.0) |
| Score Unit 3 (types and structure of simulation) | 0.84 (0.63 – 0.90) | 57 (75.0) |
| Score Unit 4 (debriefing) | 0.72 (0.38 – 0.83) | 39 (51.0) |
| Score Unit 5 (pedagogical strategy) | 0.69 (0.20 – 0.81) | 34 (45.0) |
| Weighted average score | 0.77 (0.48 – 0.81) | 51 (67.0) |

Note: Unit or weighted scores range from 0 to 1. The success threshold for the MOOC has been set at 0.7.

| **Supplementary Table 3 – Descriptive statistics of confidence and engagement variables** | | |
| --- | --- | --- |
| **Variable** | **N** | **Median (P25 – P75)** |
| Confidence Unit 1 (introduction to medical simulation) | 105 | 4.0 (4.0 – 5.0) |
| Confidence Unit 2 (errors and human factors analysis) | 105 | 4.0 (3.7 – 4.7) |
| Confidence Unit 3 (types and structure of simulation) | 105 | 4.0 (4.0 – 4.7) |
| Confidence Unit 4 (debriefing) | 104 | 4.0 (3.7 – 4.4) |
| Confidence Unit 5 (pedagogical construction) | 105 | 4.0 (3.8 – 4.5) |
| Overall confidence | 104 | 4.0 (3.7 – 4.5) |
| Emotional engagement | 107 | 4.0 (3.6 – 4.5) |
| Cognitive engagement | 107 | 3.9 (3.3 – 4.4) |
| Behavioral engagement | 107 | 4.0 (3.8 – 4.6) |
| Overall engagement | 107 | 4.0 (3.4 – 4.4) |

Note: Unit or overall confidence and engagement scores range from 1 to 5.

| **Supplementary Table 4 – Spearman’s correlations between engagement variables (N=107)** | | |
| --- | --- | --- |
|  | Cognitive engagement | Behavioral engagement |
| Emotional engagement | r_S_ = 0.80  **p < 0.001**  N = 107 | r_S_ = 0.65  **p < 0.001**  N = 107 |
| Cognitive engagement |  | r_S_ = 0.70  **p < 0.001**  N = 107 |

*r_S_: Spearman’s correlation coefficient*

| **Supplementary Table 5 – Associations between confidence and grades / engagement** | | | | |
| --- | --- | --- | --- | --- |
|  |  | **N** | **Spearman’s correlation** | **p-value** |
| Confidence Unit 1 | Grade Unit 1 | 75 | 0.07 | 0.535 |
| Confidence Unit 2 | Grade Unit 2 | 75 | -0.07 | 0.573 |
| Confidence Unit 3 | Grade Unit 3 | 75 | 0.28 | **0.014** |
| Confidence Unit 4 | Grade Unit 4 | 74 | 0.33 | **0.004** |
| Confidence Unit 5 | Grade Unit 5 | 75 | 0.22 | 0.063 |
| Overall confidence | Weighted average grade | 74 | 0.19 | 0.111 |
| Overall confidence | Overall engagement | 104 | 0.63 | **< 0.001** |

| **Supplementary Table 6 – Influence of sociodemographic variables and a priori perception of simulation on engagement variables (multivariate linear regressions) (N=103)** | | | | | | | | |
| --- | --- | --- | --- | --- | --- | --- | --- | --- |
| **Explanatory variables** | **Emotional engagement**  **(N=103)** | | **Cognitive engagement**  **(N=103)** | | **Behavioral engagement**  **(N=103)** | | **Overall engagement**  **(N=103)** | |
|  | **β** | **p-value** | **β** | **p-value** | **β** | **p-value** | **β** | **p-value** |
| Age (ref. ≤ 30) |  |  |  |  |  |  |  |  |
| 31-40 | 0.65 | **0.012** | 0.34 | 0.197 | 0.22 | 0.390 | 0.45 | **0.048** |
| > 40 | 0.56 | **0.019** | 0.31 | 0.211 | 0.27 | 0.254 | 0.41 | 0.053 |
| Origin (ref. Belgium) |  |  |  |  |  |  |  |  |
| Europe (outside Belgium) | -0.06 | 0.741 | -0.13 | 0.495 | -0.05 | 0.794 | -0.09 | 0.586 |
| Outside Europe | -0.12 | 0.620 | -0.11 | 0.657 | 0.16 | 0.523 | -0.05 | 0.827 |
| Sex (ref. female) |  |  |  |  |  |  |  |  |
| Male | 0.13 | 0.355 | 0.09 | 0.519 | -0.10 | 0.457 | 0.07 | 0.565 |
| Professional status (ref. Student) |  |  |  |  |  |  |  |  |
| Professionally active | -1.10 | **< 0.001** | -0.82 | **0.005** | -0.39 | 0.161 | -0.84 | **< 0.001** |
| Student and professionally active | -0.96 | **0.002** | -0.86 | **0.007** | -0.31 | 0.311 | -0.79 | **0.004** |
| Area (ref. Medical) |  |  |  |  |  |  |  |  |
| Paramedical | 0.08 | 0.624 | -0.05 | 0.767 | -0.08 | 0.646 | 0.00 | 0.983 |
| Education | 0.15 | 0.470 | 0.12 | 0.600 | 0.06 | 0.795 | 0.11 | 0.564 |
| Other | -0.47 | **0.030** | -0.36 | 0.103 | -0.18 | 0.399 | -0.36 | 0.057 |
| Simulation experience (ref. Yes) |  |  |  |  |  |  |  |  |
| No | 0.13 | 0.399 | 0.14 | 0.410 | 0.14 | 0.401 | 0.14 | 0.335 |
| A priori perception of simulation | 0.10 | **0.041** | 0.20 | **< 0.001** | 0.15 | **0.005** | 0.15 | **0.001** |

β: regression coefficient

| **Supplementary Table 7 – Influence of socio-demographic data, overall engagement, and a priori perception of simulation on scores (multivariate beta regression, N=73)** | | |
| --- | --- | --- |
| **Explanatory variables** | **β** | **p-value** |
| Age (ref. ≤ 30) |  |  |
| 31-40 | -0.46 | 0.375 |
| > 40 | -0.26 | 0.579 |
| Origin (ref. Belgium) |  |  |
| Europe (outside Belgium) | -0.19 | 0.603 |
| Outside Europe | -0.28 | 0.560 |
| Sex |  |  |
| Male | -0.24 | 0.391 |
| Professional status (ref. Student) |  |  |
| Professionally active | 0.32 | 0.617 |
| Student and professionally active | 0.13 | 0.842 |
| Area (ref. Medical) |  |  |
| Paramedical | -0.27 | 0.394 |
| Education | -0.38 | 0.356 |
| Other | -0.75 | 0.113 |
| Simulation experience |  |  |
| No | 0.22 | 0.474 |
| A priori perception of simulation | -0.05 | 0.583 |
| Overall engagement | 0.02 | **0.046** |

β: regression coefficient

**Supplementary Figure 1:** Changes in perception of the importance of simulation before and after completing the MOOC (n=103). (The perception of simulation was the sum of the perception of the importance of simulation in the education of healthcare professionals and of perception of the importance of simulation in the healthcare system)
